# Magnetic and electric properties of stoichiometric BiMnO_3_ thin films

**DOI:** 10.1186/s11671-015-0759-9

**Published:** 2015-02-06

**Authors:** Bo Wha Lee, Pil Sun Yoo, Vu Binh Nam, Kirstie Raquel Natalia Toreh, Chang Uk Jung

**Affiliations:** Department of Physics, Hankuk University of Foreign Studies, 81 Oedae-ro, Mohyeon-myeon, Cheoin-gu, Yongin, 449-791 Korea

**Keywords:** BiMnO_3_, Multiferroic, Ferroelectric, Stoichiometric, Centrosymmetric, 75.85. + t, 77.80.Dj, 75.70.Ak, 75.60.Ej, 81.15.Fg, 77.55.Nv

## Abstract

It has been suggested that BiMnO_3_ is a material exhibiting both ferromagnetism and ferroelectricity. Stoichiometry is rather easily achieved in a polycrystalline sample, and ferromagnetic properties have been well documented for bulk samples. Stoichiometry in thin films has been difficult to obtain, and many physical properties have exhibit wide distributions mainly due to the stoichiometry problem. Thin film studies on BiMnO_3_ have not shown clear evidence of ferroelectricity, while other physical properties measured for the BiMnO_3_ films showed wide spectra, which has been attributed to cation and/or oxygen vacancies. We fabricated BiMnO_3_ thin films with good stoichiometry and with ferromagnetic properties comparable to those reported for stoichiometric BiMnO_3_: *Tc* ~ 105 K and *M*_sat_ ~ 3.6 μ_B_/Mn. The charge-electric field (Q-E) curve measured at 5 K was fairly linear and free from hysteresis and showed no ferroelectric order. This finding is consistent with the centrosymmetric crystal structure recently suggested by theoretical calculations and structural studies on ceramic samples of stoichiometric BiMnO_3_.

## Background

BiMnO_3_ has received huge interest due to the possibility of coexistence of ferroelectricity and ferromagnetism [1-11]. BiMnO_3_ has monoclinic symmetry with lattice parameters *a* = 9.533 Å, *b* = 5.606 Å, *c* = 9.854 Å, and *β* = 110.667°. The ferromagnetism has been explained in terms of orbital ordering of Mn^4+^ ions, while the Bi-6s lone pair was expected to result in ferroelectricity. The existence of ferromagnetism has been confirmed for both stoichiometric and ceramic BiMnO_3_. Most studies of ceramic BiMnO_3_ showed around the same ferromagnetic transition temperature of approximately 105 K, with a saturated magnetic moment of 3.6 μ_B_/Mn, consistent with the high spin configuration of the Mn^4+^ ion. However, ferroelectricity of ceramic samples has not been measured. A sizable single crystal has not been obtained, and most measurements have been performed on polycrystalline samples requiring high-pressure synthesis.

It has been very difficult to obtain good stoichiometry in thin film BiMnO_3_, and the physical properties measured for thin films of BiMnO_3_ having unsatisfactory stoichiometry are often widely distributed. Thin film studies on BiMnO_3_ have not detected ferroelectricity very clearly, while other physical properties measured for the films varied across a wide spectrum. The films also exhibited non-optimum magnetic properties. Ferromagnetic transition temperatures and saturated magnetic moments were smaller than those reported for stoichiometric ceramic BiMnO_3_; thus, multiferroicity has not yet been accurately ascertained for *stoichiometric* BiMnO_3_.

The first thin film of BiMnO_3_ on SrTiO_3_ (001) substrate had *Tc* ~ 105 K, and an x-ray diffraction rocking curve peak had full width at half maximum of approximately 1.1° [12]. In that study, no measurement was made of saturated magnetic moment, *M*_sat_, and the existence of ferroelectricity was not confirmed. Son et al. reported writing polarization bits on BiMnO_3_ thin films with a low *Tc* of approximately 50 K and full width at half maximum of approximately 0.4° [13]. Pt/SrTiO_3_/BiMnO_3_/SrTiO_3_/Pt and SrRuO_3_/SrTiO_3_/BiMnO_3_/SrTiO_3_/SrRuO_3_ capacitors were also reported to show good ferroelectric properties with a remnant polarization of around 9 to 16 μC/cm^2^ but in combination with a very small saturated magnetic moment, *M*_sat_ < 1.0 μ_B_/Mn [14]. It is notable that ferroelectricity was reported to arise from SrTiO_3_ itself [15-17]. A clear polarization electric field hysteresis curve was observed for a BiMnO_3_/SrTiO_3_ (001) structure grown using pulsed laser deposition with a high Bi-rich target of Bi_2.4_MnO_3_ [18]. However, the ferromagnetic properties of the film were not optimum: *Tc* ~ 85 K, *M*_sat_ ~ 1 μ_B_/Mn. Ferromagnetic properties measured for BiMnO_3_ films made using chemical solution deposition or rf-magnetron sputtering were less favorable compared to those of stoichiometric ceramic BiMnO_3_ [19,20]. The depression in Curie temperature can be attributed to a non-stoichiometric composition, to strain, or to size effects [1].

Overall, the growth of BiMnO_3_ thin films with correct stoichiometry, free from vacancies, and with ferromagnetic properties similar to those measured in bulk samples has not yet been reported. Thin film growth of BiMnO_3_ suffers from high Bi volatility. To study the pertinent problem of multiferroicity in BiMnO_3_, we fabricated thin films of BiMnO_3_ with magnetic properties and stoichiometry matching those reported for high-pressure fabricated *stoichiometric* BiMnO_3_. Using these films, we investigated the existence of ferroelectricity in stoichiometric BiMnO_3_.

## Methods

We fabricated BiMnO_3_ thin films on a SrTiO_3_ (001) substrate using a pulsed laser deposition method [21-24]. A KrF excimer laser with repetition rate of 4 Hz was used, and the optimum growth temperature was found to be very narrow: around approximately 500°C with oxygen partial pressure of approximately 10 mTorr. We used a freshly ground surface of Bi_1.2_MnO_3_ as the target. Note that the Bi overstoichiometry is rather small; together with the precise growth conditions, these characteristics of the target are one reason for the wide spectrum of physical properties reported in films. The number of pulses required per monolayer of BiMnO_3_ was about 13.6. The thickness was estimated to be around *t* ~ 88 nm, using field emission scanning electron microscope. We performed a detailed x-ray diffraction study of the epitaxial structure of the BiMnO_3_ films using high-resolution x-ray diffraction. For electrical transport studies, we used a physical property measurement system (Quantum Design, PPMS, San Diego, USA). Magnetic properties were determined using a superconducting quantum interference device (Quantum Design, MPMS, San Diego, USA). Ferroelectric characterization measurement with capacitance geometry was done on Nb-doped SrTiO_3_ substrate\BiMnO_3_\Au sample using a cryogenic probe station (Lake Shore Cryotronics, Inc., Westerville, USA) and semiconductor parameter analyzer (Agilent Technologies, Santa Clara, USA). The area of Au top electrode was approximately 100 μm × 100 μm.

## Results and discussion

**Figure 1:**
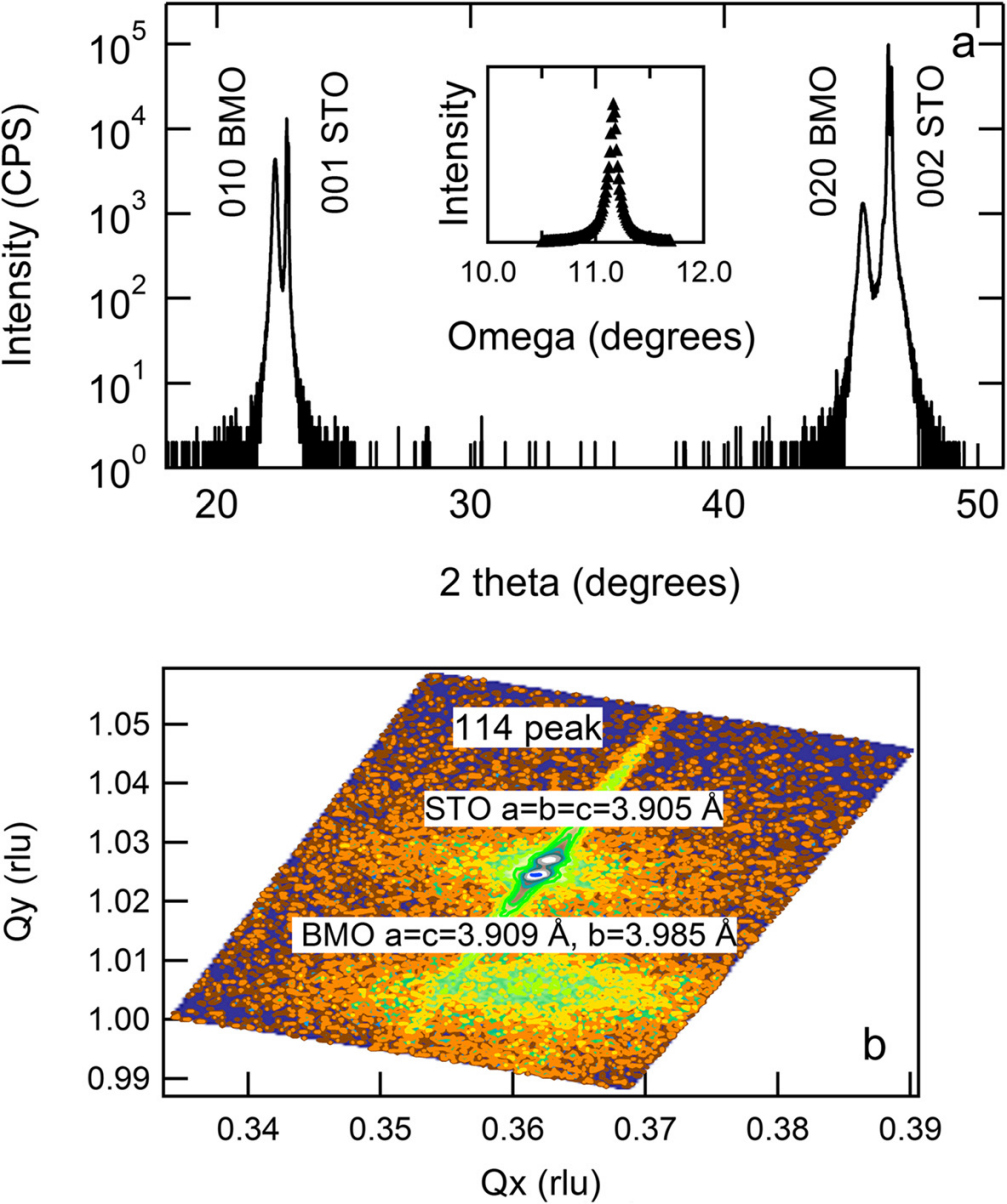
**The** ***θ*** **− 2** ***θ*** **patterns of the BiMnO**_**3**_ **/SrTiO**_**3**_ **(001) structure and the reciprocal space maps. (a)** XRD *θ* − 2*θ* patterns for the BiMnO_3_/SrTiO_3_ (001) heterostructure. Inset shows the rocking curve for a (010) BiMnO_3_ peak. The x-ray rocking curve of the (010) BiMnO_3_ peak revealed a full width at half maximum as small as approximately 0.067°. **(b)** X-ray reciprocal space mapping around the SrTiO_3_ (114) plane shows well-developed peaks for BiMnO_3_ in the lower region and two strong substrate peaks in the upper region.

Figure 1a shows the *θ* − 2*θ* patterns of the BiMnO_3_/SrTiO_3_ (001) structure. The (010) and (020) BiMnO_3_ reflection peaks are clearly visible to the left of the SrTiO_3_ substrate peaks. The calculated out-of-plane lattice constant for BiMnO_3_ film peaks was 3.985 Å. No other Bragg diffraction peaks were observed for the films. The x-ray rocking curve of the (010) BiMnO_3_ peak revealed a full width at half maximum as small as approximately 0.067°, lower than the 0.4° and 1.1° reported in previous studies [12,13].

The reciprocal space maps shown in Figure 1b confirm nearly coherent growth of a BiMnO_3_ film on an SrTiO_3_ (001) substrate with an in-plane lattice constant of 3.909 Å. The calculated volume of one unit cell of BiMnO_3_ (*V*_film_ = 60.89 Å^3^) in the film was 98.9% that of bulk monoclinic BiMnO_3_ (*V*_bulk_ = 61.58 Å^3^). The roughly 1% volume reduction is mostly due to compressive strain from the SrTiO_3_ substrate. The slightly smaller unit cell volume measured for the film demonstrates that our *stoichiometric* BiMnO_3_ has negligible cation or oxygen vacancies.

**Figure 2:**
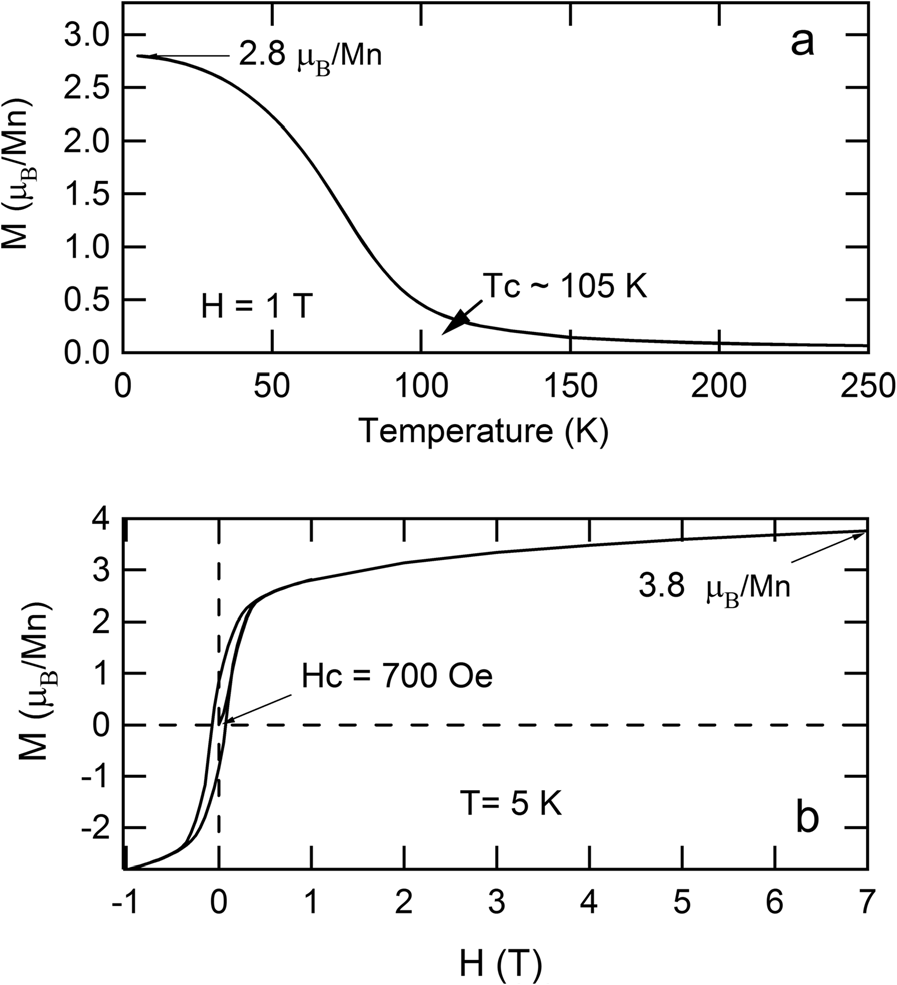
**Magnetization curves and magnetic hysteresis curves for BiMnO**_**3**_ **/SrTiO**_**3**_ **(001) structure. (a)** Magnetization curves for BiMnO_3_/SrTiO_3_ (001) structure at 1 T after 7-T high field cooling. **(b)** Magnetic hysteresis curves for BiMnO_3_/SrTiO_3_ (001) structure at 5 K.

The ferromagnetic properties for our *stoichiometric* BiMnO_3_ films were investigated, to compare their performance with that reported for stoichiometric ceramic bulk BiMnO_3_. Figure 2a shows the temperature dependence of magnetization at 1 T after 7 T field cooling. We measured a *Tc* of 105 K, close to that of the stoichiometric BiMnO_3_ bulk sample. Figure 2b shows magnetic hysteresis (*M-H*) curves at 5 K. The saturated magnetic moment is as high as 3.8 μ_B_/Mn, close to that reported for stoichiometric BiMnO_3_ bulk. The magnetic coercive field was approximately 700 Oe, which is slightly larger than that measured for other manganese perovskite oxides such as (La,Ca,Sr)MnO_3_, and about two orders of magnitude smaller than that reported for SrRuO_3_ [24,25]. For ferromagnetic perovskite oxides, a larger epitaxial strain usually results in enhancement of magnetic coercive field and the lattice mismatch of BiMnO_3_ with respect to the SrTiO_3_ substrate is larger than the lattice mismatch of (La,Ca,Sr)MnO_3_ with respect to the commonly used of SrTiO_3_ substrate.

**Figure 3:**
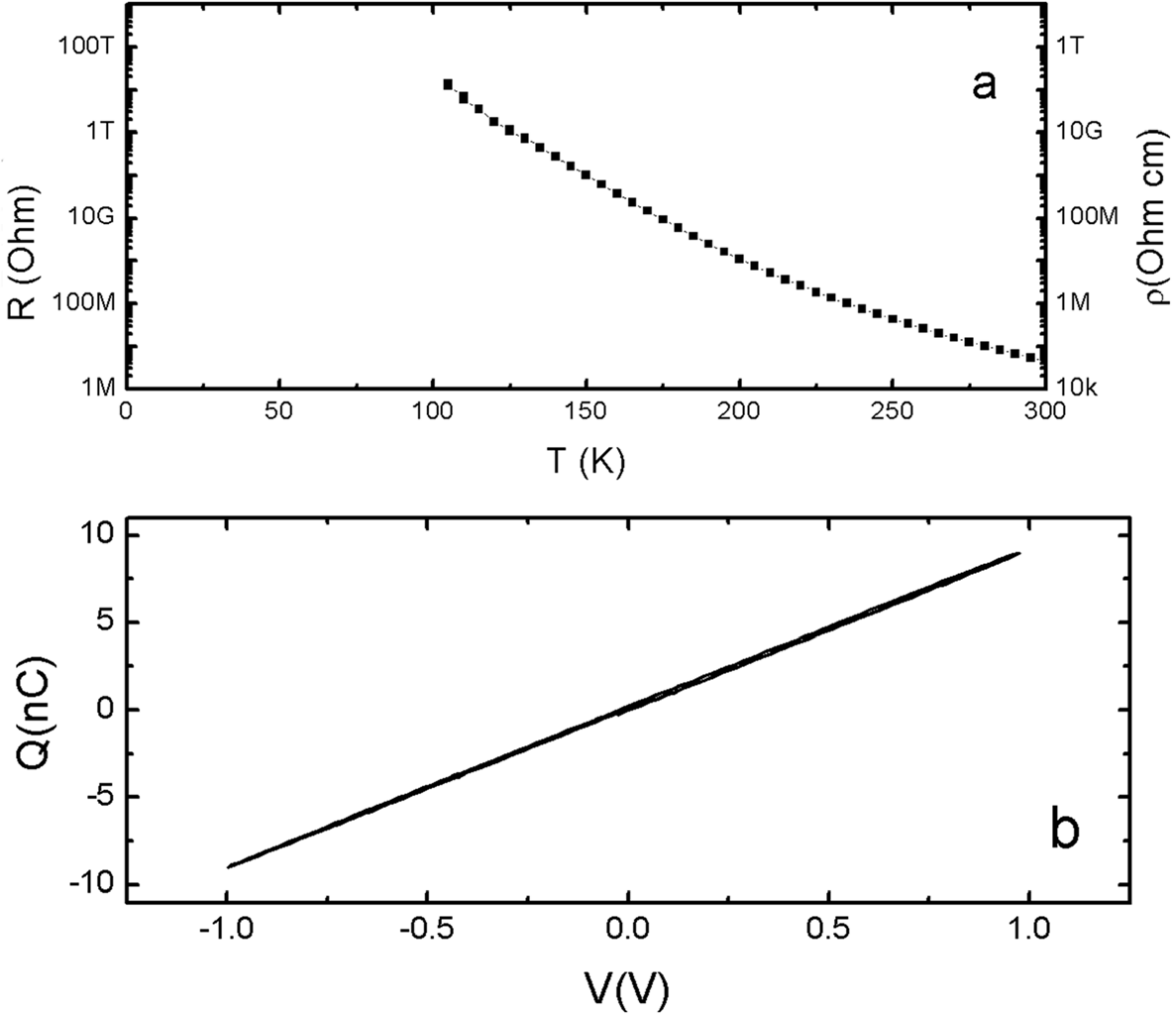
**Temperature dependence of resistivity and charge-electric field (Q-E) curve for BiMnO**_**3**_ **/SrTiO**_**3**_ **(001) structure. (a)** Temperature dependence of resistivity for BiMnO_3_/SrTiO_3_ (001) structure. **(b)** Charge-electric field (Q-E) curves for BiMnO_3_/SrTiO_3_ (001) structure at 5 K.

After confirming good stoichiometry and ferromagnetic properties, comparable to those of *stoichiometric* bulk BiMnO_3_, we investigated the existence of multiferroicity in our BiMnO_3_ films. First, in-plane resistivity was measured, as shown in Figure 3a. The resistivity of the film shows semiconducting behavior with *ρ*(*T* = 300 K) ~ 4 × 10^4^ Ω⋅cm and *ρ*(*T* = 100 K) ~ 10^11^ Ω⋅cm. This room temperature resistivity is larger than the room temperature values of 2 × 10^4^ Ω⋅cm measured for polycrystalline ceramics [5] and 1.8 × 10^2^ Ω⋅cm reported for epitaxial films [26]. However, this value is much smaller than the 5 × 10^7^ Ω⋅cm measured for a ‘*highly resistive film*’ [27]. It is notable that the out-of-plane lattice parameter of 4.004 Å reported for the ‘*highly resistive film*’ is significantly larger than our value of 3.985 Å, and that the saturated magnetic moment of 2.0 μ_B_/Mn for the ‘*highly resistive film*’ is much smaller than our value of 3.8 μ_B_/Mn. Usually, a larger unit cell volume in perovskite-based metal oxides arises from cation or oxygen vacancies, which dramatically change transport properties more than magnetic properties [28]. Gajek *et al.* demonstrated spin filtering in the BiMnO_3_ junction [26] and observed that significant changes of unit cell volume measured in films arise from Bi vacancies that locally disturb the complex orbital ordering essential for long-range ferromagnetic order in BiMnO_3_. A change of unit cell volume was accompanied by small room temperature resistivity values and lower saturated magnetic moment in the magnetic hysteresis curve [26].

Finally, we measured the charge-electric field (Q-E) curve to obtain evidence of ferroelectricity in the *stoichiometric* BiMnO_3_ film. Figure 3b shows Q-E curves at 5 K, where leakage problems, as shown in Figure 3a, do not occur. The Q-E curve measured at 5 K was fairly linear and free from hysteresis, and no ferroelectric order was observed. The absence of ferroelectric order in our *stoichiometric* film seems to be strengthened by the observation that the unit cell volume supports stoichiometry, that crystallinity is excellent, and ferromagnetic properties are as good as those reported for *stoichiometric* BiMnO_3_ samples.

Recently, there has been doubt about observations of ferroelectricity in some BiMnO_3_ films. It was reported theoretically that the ground state for BiMnO_3_ either with or without strain should be a centrosymmetric structure [29,30]. Rigorous structural studies on ceramic samples using transmission electron microscope and neutron diffraction data showed that BiMnO_3_ crystallizes in the centrosymmetric space group C2/c at 300 K [31]. It was suggested that the weak ferroelectric polarizations measured on BiMnO_3_ samples originated from an ordered oxygen deficiency [32].

## Conclusions

In summary, we investigated the existence of ferroelectricity in *stoichiometric* BiMnO_3_. We produced high-quality thin films with good stoichiometry and with magnetic properties - such as *Tc* and saturated magnetic moment - comparable to those reported for bulk *stoichiometric* BiMnO_3_. The structural quality was evidenced by narrow full width at half maximum for XRD peaks and good reciprocal space mapping data. Since vacancies in perovskite oxide film affect transport properties more than ferromagnetic properties, we believe that our *stoichiometric* BiMnO_3_ films should have sufficient quality for ascertaining the existence of ferroelectricity in *stoichiometric* BiMnO_3_. We found that the resistivity of the film demonstrates semiconducting behavior, with *ρ*(*T* = 300 K) ~ 4 × 10^4^ Ω⋅cm. The Q-E curve measured at 5 K was fairly linear and free from hysteresis, and no ferroelectric order was observed. This finding is consistent with the centrosymmetric crystal structure recently suggested by theoretical calculations and structural studies on ceramic samples of *stoichiometric* BiMnO_3_. If ferroelectricity does exist in both stoichiometric BiMnO_3_ and non-stoichiometric BiMnO_3_, then Bi-6s lone pair scenario should be the best answer for the origin. Summarizing our work and other works, the existence of ferroelectricity seems to depend on the stoichiometry very sensitively. Then, other origin should be considered at least together since Bi-6s lone pair exists both for stoichiometric BiMnO_3_ without showing FE and non-stoichiomeric BiMnO_3_ showing FE.
